# The Role of Experiential Avoidance and Parental Control in the Association Between Parent and Child Anxiety

**DOI:** 10.3389/fpsyg.2019.00262

**Published:** 2019-02-18

**Authors:** Lisa-Marie Emerson, Claire Ogielda, Georgina Rowse

**Affiliations:** ^1^School of Applied Psychology, Griffith University, Gold Coast, QLD, Australia; ^2^Clinical Psychology Unit, Department of Psychology, The University of Sheffield, Sheffield, United Kingdom

**Keywords:** parent, child, anxiety, parental control, experiential avoidance

## Abstract

Parenting behavior and practices contribute to the intergenerational relationship between parent and child anxiety, with parental control being a consistent predictor of child anxiety. Parental experiential avoidance refers to how a parent copes with their internal world in the context of parenting. Little is known about how this relatively new parenting concept relates to child anxiety. The current study tested the indirect effect of parent anxiety on child anxiety through parental control and parental experiential avoidance; the indirect effect of parent anxiety on parental control through parental experiential avoidance; and the moderating effect of parental experiential avoidance on the relationship between parental control and child anxiety. Using a cross-sectional design, parents (*N* = 85) from a community sample of 8–12-year-old children self-reported on a survey measuring parent anxiety, child anxiety, parental control, and parental experiential avoidance. A hierarchical regression indicated that parental experiential avoidance significantly predicted child anxiety and accounted for further variance in child anxiety, over, and above parental control. There was an indirect effect of parent anxiety on child anxiety through parental control and parental experiential avoidance. Parental experiential avoidance moderated the relationship between parental control and child anxiety, such that the relationship was only significant at high levels of parental experiential avoidance. The current study provides support for the role of parental experiential avoidance in an intergenerational understanding of anxiety. Future research should replicate the study with a clinical sample. Theoretical and practice implications are considered.

## Introduction

Fear, worry, and anxiety are common during childhood and for most children occur as part of normal development. However, for some children anxiety symptoms become worse over time and interfere with daily routine and interpersonal functioning (Breinholst et al., 2012). Anxiety has been found to be one of the most common psychiatric problems in children and adolescents (Costello et al., 2005) and Beesdo et al. (2009) reported a prevalence of up to 15–20% of children experience some level of anxiety at one time. Children have been found to experience anxiety at all stages of childhood; however, middle childhood (8–12 years) is a common time for children to present to services with anxiety.

Research has consistently identified that parental anxiety is a risk factor for childhood anxiety (Donovan and Spence, 2000); children of parents who have an anxiety disorder are five to seven times more likely to be diagnosed with an anxiety disorder themselves compared to children of parents who do not have an anxiety disorder (Beidel and Turner, 1997). The co-occurrence of parental and child anxiety has led many researchers to suggest that anxiety is transmitted from parent to child (Remmerswaal et al., 2015). Burnstein and Ginsburg (2010) suggested that this transmission is partially genetic; with genetic heritability accounting for approximately 50% of the variance in children having an anxious disposition (Eley and Gregory, 2004). The heredity of anxiety disorders, more specifically, is estimated to be lower (Kendler et al., 1992). Given that genetic heredity cannot account for all the variance in child anxiety, previous research has also explored the influence of parental characteristics such as parental attachment (Brumariu and Kerns, 2010) and parenting style (Waite et al., 2014), as explanatory factors.

Craske (1999) model of anxiety development postulates that parenting style provides an environmental context that can influence the development and maintenance of anxiety. For example, a relationship between child anxiety and high levels of parental rejection and parental control has been found (Bőgels and Brechman-Toussant, 2006; van der Sluis et al., 2015). Several systematic literature reviews indicate that high levels of control in parenting is the most consistent parenting predictor of anxiety in childhood (Ballash et al., 2006; McLeod et al., 2007; Murray et al., 2009), accounting for around 6% of the variance in child anxiety (McLeod et al., 2007).

Parental control is characterized by excessive monitoring of their children’s activities, discouragement of independent thinking and high levels of intrusion (Bőgels and Brechman-Toussant, 2006). Traditionally, parental control has been defined both in terms of behavioral control and psychological control of the child (Barber, 1996). Parental control of the child’s behavior can involve imposition of structure, expectations and contingencies; whereas psychological control refers to a pattern of manipulating and restricting the child’s emotional experience. With this classical distinction, previous literature has indicated that mothers of anxious-withdrawn children are both more behaviorally and psychologically controlling than mothers of average children (Mills and Rubin, 1998). Within inter-generational theories of child anxiety development, the idea of behavioral and psychological control are combined in relation to parental rearing behavior, with parental control defined as parental behavior that limits or threatens the child’s autonomy (Grusec and Davidov, 2007). Parental control may take the form of both psychological or behavioral in nature, with the aim of constraining the child’s cognition and emotion (Borelli et al., 2015). In their meta-analysis, McLeod et al. (2007) classified parental control into two subdimensions. The first, over-involvement was defined by parent interference with normative autonomy of the child, excessive restriction, and encouragement of dependence. The second subdimension was low autonomy granting, which referred to parental discouragement of the child’s opinions and input in decision-making. Collectively, over-involvement and autonomy granting accounted for the largest proportion of variance in child anxiety compared to other parenting factors, such as warmth. McLeod et al. (2007) reported a larger effect size in the relationship between child anxiety and parental (low) autonomy granting (0.42) in comparison to parental over-involvement (0.23). In this sense, parental control has been viewed as parental overprotection and low autonomy granting (Ollendick and Grills, 2016). Chorpita and Barlow (1998) suggest that parental control can lead to a vulnerability to child anxiety due to a reduction in the child’s development of autonomy. Barlow (2002) model of anxiety development suggests that perceived lack of external and internal control is an important attribute in the development of anxiety for both adults and children. Consistent with this theoretical assertion, Thirlwall and Creswell (2010) reported that children experienced greater levels of anxiety when their parents behaved in controlling ways, compared to autonomy granting ways.

Despite the consistent link between parental control and child anxiety (Murray et al., 2009), the link between parent anxiety and parental control is less clear. There is empirical support for a link between parent anxiety and controlling parenting behavior (Whaley et al., 1999), but in a meta-analytic review of 23 studies, van der Bruggen et al. (2008) reported a non-significant relationship between parent anxiety and parental control. Further, Turner et al. (2003) found no difference between anxious and non-anxious parents in levels of parental control. Therefore, there is inconsistent evidence that parents who experience higher levels of anxiety will be more likely to engage in controlling behaviors.

More recently, researchers have investigated whether parental control is a mechanism that could explain the relationship between parent and child anxiety, with mixed results. Affrunti and Woodruff-Borden (2015) did not find that parental control mediated the relationship between parent and child anxiety. Conversely, Borelli et al. (2015) found that maternal control mediated the relationship between maternal anxiety and child anxiety; however, paternal control was not found to mediate the relationship between paternal anxiety and child anxiety. One possible explanation for the inconsistent finding, may be that there is another parenting factor unaccounted for within this relationship. Tiwari et al. (2008) postulated that parents may engage in controlling behaviors as a means of avoiding their own internal distress; in this sense, parental control is viewed as a manifestation of parental experiential avoidance.

Experiential avoidance refers to the inability or unwillingness to remain in contact with ones’ own internal distress (Heckler, 2012, Unpublished). In finding ways of regulating emotional distress, one may engage in behaviors or strategies to suppress, avoid or escape these feelings. Experiential avoidance has been found to be important in both the development and maintenance of anxiety in both adults and children (Simon and Verboon, 2016). Given that anxious adults are likely to engage in experiential avoidance (Barman et al., 2010); it follows that parents who are anxious may also engage in parenting specific experiential avoidance.

An anxious parent may deal with difficult parenting experiences that lead to their own internal distress by avoiding, suppressing, or controlling (Tiwari et al., 2008) as a means to relieve their own distress. One response may be to intervene, for example, by removing their child from the situation. Subsequently, the child is not afforded the opportunity to engage in ‘trial and error’ learning which leads to the development of self-efficacy (Cartwright-Hatton et al., 2010), and so may become vulnerable to anxiety. Consistent with this supposition, Hudson et al. (2008) reported that mothers of anxious children behaved intrusively when their child displayed negative emotions, compared to when the child displayed positive emotions. The authors suggest that parents may have felt uncomfortable when their child expressed negative affect and because of this, were driven to behave in controlling ways to reduce their own discomfort as well as their child’s negative emotions.

To date, only one study has examined the relationship between parental experiential avoidance and child anxiety; Cheron et al. (2009) reported that parental experiential avoidance was significantly associated with high levels of child anxiety in a sample of children with anxiety disorder. In addition, parents who reported high levels of experiential avoidance in their daily lives, also reported high levels of experiential avoidance in their parenting style and were more likely to experience anxiety themselves.

The current study aimed to examine the predictive relationships between child anxiety and two theoretically related parenting factors: parental control and parental experiential avoidance and to explore these parenting dimensions within the relationship between parent and child anxiety. Given inconsistencies reported in previous research, two clear objectives were to (i) investigate the indirect effect of parent anxiety on child anxiety through parental control and parental experiential avoidance and (ii) the indirect effect of parent anxiety on parental control through parental experiential avoidance. Finally, we aimed to investigate if the relationship between parental control and child anxiety would be moderated by parental experiential avoidance.

## Materials and Methods

### Participants

Parents of children aged between 8 and 12 years, who were the main caregiver and had sufficient proficiency in English, were invited to take part in the study. A total of 120 questionnaires were distributed to a community sample; 85 parents returned completed measures. The sample of participants comprised 12 fathers and 73 mothers, who reported on their children (38 boys, 47 girls) with a mean age of 9.83 years (*SD* = 1.28). The majority of the sample of participants identified themselves as White British (80%); the remaining participants identified as either Irish (2.4%), American (2.4%), Asian (1.2%), black Caribbean (3.5%), Indian (2.4%), black British (3.5%), white European (3.5%) or did not specify (1.2%). No participants were below 25 years in age; 9.4% of parents were between ages 26–35, 61.2% of parents were between ages 36–45 and 29.4% of parents were over 46. Parents reported a range in average annual household income; 11.8% reported less than £20,000, 7.1% reported £20–30,000, 17.7% reported £30–50,000, 32.9% reported £50–70,000, 16.5% reported £70–100,000 and 14.1% reported over £100,000.

### Procedure

The study protocol was approved by the National Health Service, United Kingdom, research ethics committee. Study advertisements were distributed to four local primary schools and two community groups, and directed parents to collect a paper version of the study information sheet and questionnaire from the reception area if they were interested in participating. Participants completed questionnaires at their leisure, and returned completed questionnaires in a sealable envelope (provided with the questionnaire pack) via a dedicated covered box left in the respective reception area. Anonymity was retained; participants were not required to provide any personally identifying information about themselves or their child. The return of questionnaires was considered as implied consent.

Participants with more than one child between 8 and 12 years were instructed to base their responses on the child whose age was closest to the mid-range (i.e., 10 years). Parents with children who were the same age (i.e., twins), were asked to bring one of their children to mind when completing the questionnaire.

### Measures

#### Parent Rated Child Anxiety Symptoms

The symptoms were measured using the Spence Children’s Anxiety Scale, Parents Version (SCAS-P; Spence, 1998). The SCAS-P is a 39-item parent-report measure, which generates total scores and subscale scores in accordance with DSM-IV (American Psychiatric Association [APA], 2000) anxiety disorder clusters. Participants rated the degree to which their child experiences each symptom (e.g., “my child complains of feeling afraid”) on a four-point Likert scale, from never (0) to always (3). Total scores range from 0 to 114; higher scores indicated higher levels of child anxiety. Norms for mean total scores of children with an anxiety disorder range from 30.1 (*SD*: 14.9) to 33.0 (*SD*: 14.9) and in the community sample, norm mean total scores range from 11.8 (*SD*: 8.3) to 16.0 (*SD*: 11.6) in children aged between 6 and 18 years (Nauta et al., 2004). The SCAS-P has demonstrated good validity and reliability with an overall Cronbach’s alpha coefficient of 0.89 and consistency has been found between child and parent versions. In the current study, the Cronbach’s alpha coefficient was 0.96.

#### Parent Anxiety Symptoms

The symptoms were measured using the State Trait Anxiety Inventory (STAI, Spielberger et al., 1983). The STAI is a 40-item self-report questionnaire, which includes two subscales measuring both state and trait levels of anxiety. For the purpose of this study, the trait subscale was used to measure parent’s anxiety, as an indicator of more enduring levels of anxiety. The trait anxiety subscale has 20 items referring to symptoms of anxiety (e.g., “I feel nervous and restless”); participants indicated how often they experience each symptom on a four-point Likert scale from 1 (almost never) to 4 (almost always). A total trait anxiety score was obtained by summing scores on the 20-items. Total scores range from 20 to 80; higher scores indicate greater levels of trait anxiety. Clinical cut-off scores have not yet been defined. However, in a sample of parents with an anxiety disorder, Teetsel et al. (2014) reported the mean total score for mothers to be 49.82 (*SD*: 8.29) and for fathers to be 49.81 (*SD*: 9.16). The STAI correlates highly with other measures of adult anxiety and has shown good test–retest reliability in other samples (*r* = 0.73 to *r* = 0.85; Spielberger et al., 1983). In the current study, the Cronbach’s alpha coefficient was 0.95.

#### Parental Control

It was measured using The University of Southern California Parental Control Scale (USC-POS, Borelli and Margolin, 2013, Unpublished). The USC-POS is a 10-item scale designed to measure behavioral, affective and cognitive aspects of parental control and child autonomy restriction (an example of a cognitive aspect assessed, “I expect my child to tell me what happens when he/she is away from home”; example of a behavioral aspect “When I am disappointed or irritated with my child, I withhold affection”; example of a parental behavior aimed at constraining their child’s thought and feelings, “I do not allow my child to get angry with me”). Participants rated each item according to how well it described their parenting, on a five-point Likert scale from 0 (not at all descriptive) to 4 (extremely descriptive). Total scores can range from 0 to 40; higher scores indicating higher levels of control used in parenting. The USC-POS has demonstrated good internal consistency (α = 0.81) and validity. In the current study, the Cronbach’s alpha coefficient was 0.88.

#### Experiential Avoidance in Parenting

It was measured using the Parental Acceptance and Action Questionnaire (PAAQ, Cheron et al., 2009). The PAAQ is a 15-item self-report measure of parent’s willingness to witness their child experiencing distress, as well as a parent’s ability to manage their reaction to their child’s distress. Item statements (e.g., “worries can get in the way of my child’s success”) were rated by participants on a seven-point Likert scale ranging from 1 (never true) to 7 (always true). Total scores can range from 15 to 105; higher scores indicate a higher degree of parental experiential avoidance. The PAAQ has demonstrated moderate internal consistency (α = 0.64–0.65) and moderate test re-test reliability (α = 0.68–0.74). In the current study, the Cronbach’s alpha coefficient was 0.83.

### Data Analyses

An *a priori* power calculation was undertaken for multiple regression analysis. Assuming a medium effect size of *R*^2^ = 0.15, a significance level of α = 0.05, and four predictor variables, a sample size of 85 participants was required to achieve 80% power.

Independent *t*-tests using bootstrapping procedures with 1000 re-samples and the bias corrected confidence interval were conducted to test for differences in parent and child gender for all measured variables. Any significant differences found in gender mean scores, were controlled for in further analysis as a covariate. As assumptions in linearity of the data could not be assumed, Spearman’s Rho correlations were conducted to test the associations between variables. Hierarchical regression analyses were performed to examine the amount of variance in child anxiety could be explained by the parenting variables. Bootstrapping tests with 1000 re-samples and the bias corrected confidence interval were performed and variables were entered into the regression model using a forced entry method.

Mediation and moderation analyses were performed using model 4 of the PROCESS macro (Hayes, 2013) for the Statistical Package for Social Sciences (SPSS). To examine indirect effects, the paths from the predictor variable to the mediator(s) (path a), the mediator(s) to the outcome variable (path b) and the predictor variable to the outcome variable (path c) were inspected for significance. If the path between the predictor variable and the outcome variable (path c′) became non-significant when controlling for mediating variable(s), a mediation effect was indicated. If this occurred, as recommended by Preacher and Hayes (2004) bootstrapping procedures were applied with 5000 re-samples and the bias corrected confidence interval to establish whether indirect effects through individual potential mediator(s) were significant.

For moderation, the interaction between parental control and parental experiential avoidance (step 4) was added to the initial hierarchical regression analysis. The contribution of the interaction term in predicting child anxiety was assessed by inspection of *R*^2^_change_, along with accompanying *F* statistic and *p*-value (<0.05 indicated significance). If potential moderation (significant interaction term) was indicated, then regression analysis on the centered terms was conducted using the PROCESS macro; bootstrapped data (5000 re-samples) were inspected and plotted, to assess the conditional effect of parental control on child anxiety, under high and low values of parental experiential avoidance.

## Results

### Data Screening

Confident assumptions of normality, linearity, and homoscedasticity could not be made for all variables. Multicollinearity was not deemed to be problematic. Violations of normality are generally not considered to be highly problematic within bootstrapping procedures, with regression analyses being robust to violations (Tabachnick and Fidell, 2013). Three individual item scores (0.04%) across the dataset were missing; these were replaced with the participant’s mean score on that measure. No outliers were indicated as influencing the overall findings. An inspection of STAI scores showed that 16 participants (19%) scored above, and 27 (32%) participants scored within one standard deviation above and below clinical norm mean scores (Teetsel et al., 2014). Inspection of the SCAS-P scores showed that 12 participants (14%) reported their child to score over the clinical norm mean score, and a total of 44 participants scored their child within one standard deviation above and below the mean norm score for clinical levels of anxiety (Nauta et al., 2004).

### Preliminary Analyses

Significantly higher child anxiety scores were observed for boys (*M* = 27.18, *SD* = 22.50) compared to girls (*M* = 14.47, *SD* = 11.93), *t*(53.51) = 2.90, *p* < 0.005, with a small effect size (*d* = 0.14). Child gender was subsequently controlled for as a covariate in subsequent regression and mediation analyses. No significant differences were observed between mothers and fathers on parent anxiety score, parental control, and parental experiential avoidance (*p* > 0.05). Means and standard deviations for each of the measures are reported in Table 1.

**Table 1:** Descriptive statistics and Spearman’s Rho correlation coefficients for all primary measures (*N* = 85).

Variable (*n* = 85)	1	2	3	4
(1) Parental anxiety (STAI)	–			
(2) Child anxiety (SCAS-P)	0.51^∗∗^	–		
(3) Parent control (USC-POS)	0.48^∗∗^	0.43^∗∗^	–	
(4) Parent experiential avoidance (PAAQ)	0.46^∗∗^	0.54^∗∗^	0.61^∗∗^	–
Range	22–75	0–76	1–31	21–89
*M* (SD)	39.27 (12.07)	20.71 (18.31)	8.88 (6.85)	50.88 (13.72)

*^∗∗^p < 0.001.*

### Correlation Analyses

A significant moderate correlation was observed between parent and child anxiety scores; parents who reported high levels of anxiety, also reported that their child experienced high levels of anxiety. A significant moderate correlation was observed between parental experiential avoidance and child anxiety; parents who reported high levels of experiential avoidance, also reported that their child experienced high levels of anxiety. Correlation coefficients are reported in Table 1.

### Regression Analyses

Results from the regression analysis are reported in Table 2. In block one, child gender and parent anxiety (STAI) explained 58% of the variance in child anxiety (SCAS-P), *R*^2^ = 0.58, *R*^2^_Adjusted_ = 0.57, *F*(2,82) = 56.97, *p* = < 0.001, with parent anxiety explaining a significant amount of the variance. The addition of parental control at block two explained a further 7% of the variance in child anxiety, Δ*R^2^* = 0.07, *R*^2^_Adjusted_ = 0.64, *F*(3,81) = 50.84, *p* < 0.001, making a significant contribution to the model. The addition of experiential avoidance at block three explained a further 2% of the variance in child anxiety, Δ*R^2^* = 0.2, *R*^2^_Adjusted_ = 0.66, *F*(4,80) = 41.20, *p* < 0.001, with parental experiential avoidance making a significant contribution to the variance. Thus the final model predicted 67% of the total variance in child anxiety. Parental control remained a significant predictor at block 3, with a reduction in beta value observed. Parent anxiety remained a significant predictor within each block and the final model, explaining the greatest proportion of variance in child anxiety, although with a decreasing beta size observed.

**Table 2:** Summary of regression analysis predicting child anxiety from child gender and parental anxiety (step 1); parental control (step 2); parental experiential avoidance (step 3).

Block	Variable	*B*	*SE*(B)	β	Confidence intervals	
					Lower	Upper
1	Child gender	−0.54	2.85	−0.02	−5.95	4.57
	Parent anxiety	1.15	0.15	0.76^∗∗∗^	0.81	1.37
2	Child gender	−0.62	2.53	−0.02	−5.34	3.86
	Parent anxiety	0.72	0.16	0.48^∗∗∗^	0.43	0.99
	Parent control	1.03	0.27	0.39^∗∗∗^	0.53	1.56
3	Child gender	−0.58	2.49	−0.02	−5.23	4.05
	Parent anxiety	0.65	0.16	0.43^∗∗∗^	0.34	0.93
	Parent control	0.69	0.31	0.26^∗^	0.13	1.26
	Experiential avoidance	0.29	0.11	0.22^∗^	0.05	0.52

*N = 85. Block 1 R^2^ = 0.58^∗∗∗^, Block 2 ΔR^2^ = 0.07^∗∗∗^, Block 3 varΔR^2^ = 0.02^∗∗∗^. ^∗^p < 0.05, ^∗∗∗^p < 0.001.*

### Indirect Effects of Parent Anxiety on Child Anxiety Through Parenting Factors

**FIGURE 1:**
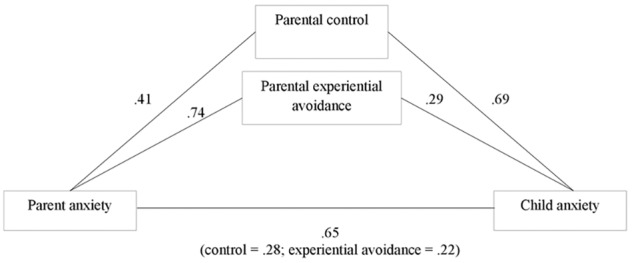
Coefficients for indirect association of parent anxiety and child anxiety via parental control and parental experiential avoidance.

The first mediation model assessed the indirect effects of parent anxiety (IV) on child anxiety (DV) via the two parenting factors, parental control, and experiential avoidance (MV). Parent anxiety was a significant predictor of parental control (path a), β = 0.41, *SE* = 0.04, *p* < 0.001, and parental experiential avoidance, β = 0.74, *SE* = 0.09, *p* < 0.001. Parental control was a significant predictor of child anxiety (path b), β = 0.69, *SE* = 0.29, *p* = 0.019. Parental experiential avoidance was a significant predictor of child anxiety (β = 0.29, *SE* = 0.13, *p* = 0.028). Parent anxiety continued to be a significant predictor of child anxiety (path c′), β = 0.65, *SE* = 0.14, *p* < 0.001. Bootstrapping procedures indicated that the indirect effects of parental control (β = 0.28, 95% BCa CI [0.03, 0.61]), and parental experiential avoidance (β = 0.22, 95% BCa CI [0.05, 0.39], were significant. See Figure 1.

**FIGURE 2:**
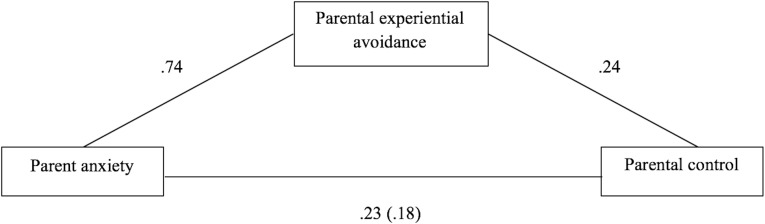
Coefficients for indirect association of parent anxiety and child anxiety via parental control and parental experiential avoidance.

The second mediation model assessed the indirect effects of parent anxiety (IV) on parental control (DV) via parental experiential avoidance (MV). Parent anxiety was a significant predictor of parental experiential avoidance (path a), β = 0.74, *SE* = 0.09, *p* < 0.001. Parental experiential avoidance was a significant predictor of parental control, β = 0.24, *SE* = 0.04, *p* < 0.001 (path b). Parent anxiety continued to be a significant predictor of parental control (path c′), β = 0.23, *SE* = 0.05, *p* < 0.001. Bootstrapping procedures indicated that the indirect effect of parental experiential avoidance, β = 0.18, 95% BCa CI [0.10, 0.25], was significant. See Figure 2.

### Parental Experiential Avoidance Moderates Relationship Between Parental Control and Child Anxiety

**FIGURE 3:**
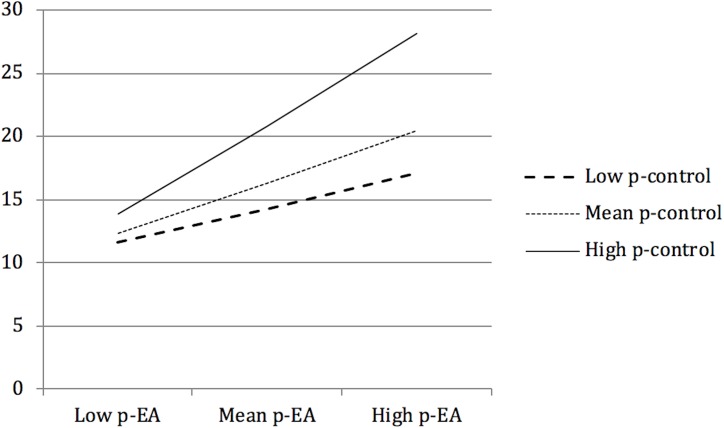
Interaction plot of child anxiety at different values of parental control (p-control) and parental experiential avoidance (p-EA).

The addition of the interaction term, parental control x parental experiential avoidance, to the hierarchical regression predicting child anxiety (step 4), resulted in a significant amount of additional variance explained, Δ*R^2^* = 0.03, *R*^2^_Adjusted_ = 0.68, Δ*F*(5,79) = 6.57, *p* = 0.012. Data for conditional effects of parental control at values of parental experiential avoidance indicated that at low values of parental experiential avoidance the relationship between parental control and child anxiety is non-significant; at high values of parental experiential avoidance, the relationship between parental control and child anxiety is significant. Examination of the interaction plot (see Figure 3) confirmed an enhancing effect; as parental experiential avoidance and control increased, so child anxiety increased. At low parental experiential avoidance, child anxiety was similar for low, average, or high levels of parental control. Those parents reporting high levels of parental experiential avoidance and high levels of parental control, also reported the highest levels of child anxiety.

## Discussion

The current study investigated the intergenerational relationship between parent and child anxiety, and examined parenting factors that may be associated with and account for the variance in child anxiety. More specifically, we extended previous lines of inquiries around the role of parental control by the inclusion of an affective parenting component, namely experiential avoidance. Parental experiential avoidance has been posited as a potential explanation for parental controlling behavior in relation to child anxiety. The two objectives of our study were to investigate: (i) the indirect effect of parent anxiety on child anxiety through parental control and parental experiential avoidance and (ii) the indirect effect of parent anxiety on parental control through parental experiential avoidance.

The findings from regression analyses indicated that parental experiential avoidance was a significant predictor of child anxiety, after controlling for parent anxiety and parental control. Consistent with previous research (Francis and Chorpita, 2011), parent anxiety was a significant predictor of child anxiety, lending further support to research that has demonstrated that anxiety co-occurs in parents and children (Waters et al., 2012). In line with our predictions, parental control emerged as a significant predictor of child anxiety, accounting for 7% of the variance in child anxiety. The amount of variance explained by parental control is consistent with that which is reported by McLeod et al. (2007) in their meta-analysis and with previously reported correlations (Murray et al., 2009). The association replicated here lends support to theories that emphasize parental control in the development and maintenance of child anxiety (Chorpita and Barlow, 1998), which suggest that when parents fail to give their child the opportunity to experience control in age appropriate contexts, the child may be vulnerable to developing anxiety (Barlow, 2002). We extended this understanding, by demonstrating that parental experiential avoidance is also a key predictor of child anxiety. Similarly, Cheron et al. (2009) also found that parental experiential avoidance predicted child anxiety.

The current study is also the first to demonstrate the indirect effects of parent anxiety on child anxiety through parental experiential avoidance. These findings indicate a key role for parental experiential avoidance in explaining the co-occurrence between parental and child anxiety, which has not previously been considered. An intolerance for their own and their child’s distress is likely to be a triggering factor in how parents behave with their child. Parents’ own cognitive and affective coping, such as experiential avoidance, may influence their parenting behavior with their child, as well as modeling to their child how a potentially ineffective means of coping with anxiety.

Tiwari et al. (2008) theorized that experiential avoidance may be a mechanism that leads parents to behave in controlling ways. Hence, in order to control, alter or avoid intolerable thoughts and feelings in relation to their child being in distress, parents may behave in controlling ways in order to diminish their own internal distress (Tiwari et al., 2008), for example engaging in high levels of intrusion or excessively monitoring their child’s activities (Bőgels and Brechman-Toussant, 2006). We demonstrated that parental anxiety predicted increased parental control, and that there was an indirect effect through parental experiential avoidance. Parents with higher anxiety are more likely to struggle to tolerate the experience of their child being in distress, and as such are more likely to engage in controlling behaviors to rid themselves of their own associated distress. In this manner, parental experiential avoidance may be the affective trigger to parents’ behavioral responses toward their child in those moments of high anxiety, or indeed to prevent such from occurring.

Moderation analyses indicated that the relationship between parental control and child anxiety was only significant under conditions of high parental experiential avoidance. When parents report high levels of experiential avoidance, higher levels of parental control are associated with higher levels of child anxiety. Therefore, as a result of parental control, the child may not learn valuable coping skills, which in turn may lead to them being vulnerable to developing anxiety (Chorpita and Barlow, 1998). Consistent with this hypothesis, Hudson et al. (2008) found that mothers of anxious children were more intrusive, a key aspect of parental control, when their child displayed negative emotions, compared to positive emotions. The interaction between parental control and experiential avoidance in the current study postulates a possible mechanism for this parental controlling and intrusive responding. The current study suggests that high levels of experiential avoidance may impair a parent’s ability to respond sensitively to situations that induce anxiety in their child (Raftery-Helmer et al., 2016). When parents report low levels of experiential avoidance, then parental control is unrelated to child anxiety. The exact mechanism of this interaction requires further investigation. As these relationships are bidirectional, it may be that increased child anxiety provokes parental control only when parents are high in experiential avoidance.

The presence of direct effects in our mediation models indicates that other mechanisms that were not assessed are involved in the relationships between the variables studied, for example parents’ own anxiety will influence their reporting of their child’s anxiety. In addition, parents’ expectations of their child will likely also impact on their reporting of their child’s anxiety. For example, parent expectation of their child’s ability to cope has been shown to be associated with the development of child anxiety (Emerson et al., 2018). Notwithstanding the contribution of other parenting factors, such as warmth, rejection, and attachment, we know that genetics account for a large proportion of variance in the association between parent and child anxiety (Eley and Gregory, 2004). In addition, demographic variables such as gender and age are likely to influence the associations observed in the current study.

Although we did not set out to examine the role of gender in our study, two findings of interest emerged from our data. First, parents of boys reported that their child experienced significantly higher levels of anxiety than parents of girls. The increased levels of anxiety reported for boys, compared to girls, contrasts with previous research, which has found that girls experience higher rates of anxiety than boys (e.g., Roza et al., 2003; Waters et al., 2012). Second, no differences were observed between reports from mothers (*n* = 73) and fathers (*n* = 12) in the current study, in relation to their own anxiety, or parenting factors (control and experiential avoidance). The unequal numbers of mothers and fathers in this study limits inferences that can be drawn from the observed findings, and precluded subsequent analyses. Previous research that has aimed to assess differences between mothers and fathers reported that women experienced greater anxiety, avoidant coping, and experiential avoidance than men (Panayiotou et al. (2017). Parental gender may interact with the observed relationships in the current study, given previous research demonstrating differential relationships between parental control and child anxiety by parent gender and child age (Verhoeven et al., 2012). The picture regarding the effects of development, gender and environment is complex and beyond the scope of this study. However, future research should seek to clarify the role of child and parent gender in the relationship between these parenting factors and child anxiety.

The current findings suggest that parental experiential avoidance should be considered alongside parental control in future research on parent and child anxiety. Targeting parental distress tolerance may be an avenue for improving treatment effects for child anxiety; however, recent research provides some inconsistent preliminary findings in this regard. Emerson et al. (unpublished) demonstrated that improvements in parental experiential avoidance following a mindfulness-based parenting intervention uniquely predicted improvements in child internalizing problems. In contrast, a pilot randomized controlled trial of a tailored parent intervention targeting parental distress tolerance, Hiller et al. (2016) reported no differences in child anxiety outcomes compared to standard behavioral parenting intervention. Hiller et al. (2016) did report differential effects on positive parenting and the quality of the parent–child relationship, with increases for those in the targeted treatment condition, and decreases for those in the standard condition. The question remains whether targeting parental experiential avoidance within parenting interventions may or may not indicate improvements in child outcomes. The effects of such an intervention may be broader, in relation to decreased negative parenting and increased positive parenting, with relational implications in the parent–child dyad.

### Limitations and Future Research

The proposed model of effects of parent anxiety on child anxiety via parental control and experiential avoidance is consistent with previous research and theory of the intergenerational relationship of anxiety. However, the current study has a number of limitations that should be noted. It is likely that the overall explanatory model of these parenting factors will be more complex than is possible to assess in the current study. For example, parents’ own experience of anxiety will influence the interaction between the two parenting factors: control and experiential avoidance. In turn this will have differential associations with child anxiety. Future longitudinal research is therefore necessary to assess a potential moderated mediation model, with the indirect effect of parental control on child anxiety via parental experiential avoidance being moderated by parental anxiety. This research necessitates child self-report, or clinician assessment of child anxiety, in order to remove potential bias created by parents’ own anxiety.

Research using longitudinal and experimental designs is also required to disentangle the direction of the relationships observed and thus clarify whether anxious children evoke parental experiential avoidance and controlling parenting or whether parental control and parental experiential avoidance develops as a response to the parent’s own anxiety. Future research should also examine other unhelpful parenting behaviors that parents may employ when they engage in experiential avoidance, which may impact on child anxiety. For example, parental rejection has also been found to be an important parenting behavior in child anxiety (Bőgels and Brechman-Toussant, 2006). Parents may be more likely to behave in rejecting ways when they are finding it difficult to tolerate their child’s distress.

The current study measured the concept of experiential avoidance in terms of parents finding it difficult to tolerate seeing their child in distress (Cheron et al., 2009). However, it is possible that parents who experience anxiety may also engage in experiential avoidance when they experience difficult emotions in relation to their child behaving in a way that may be anxiety provoking for them, but not causing any distress to the child (Tiwari et al., 2008). Therefore, future research would benefit from examining this aspect of experiential avoidance that parents may engage in, in relation to parent and child anxiety.

The current study relied on parental report of parenting behavior and child anxiety. While this is a common approach reported in the literature (Bőgels and Brechman-Toussant, 2006; Borelli et al., 2015), previous research has demonstrated that parents often under report negative parenting behavior, when compared to child reports of the same behavior (Bögels and van Melick, 2004). Furthermore, when parent and child reports of child anxiety are compared, there is often low agreement between informants (Bőgels and Brechman-Toussant, 2006). The associations observed in the current study may be unique to parents’ perceptions; their report of their child’s anxiety will likely be enmeshed with their predictions of their child’s ability to cope, which is inevitably influenced by their own experience of anxiety. Nonetheless, parent perceptions are valid and pivotal to understand given that they are usually the driving force behind support seeking and advocacy for their child. Future work should include multiple reporting with both parent and child reports and observational measures in order to disentangle the influence of parental cognitions and beliefs of their perception of their child, and aid further understanding of the observed relationships in the current study.

Further, in relation to measurement, the parenting constructs assessed in the current study are likely to have a degree of overlap. The correlation coefficients did not preclude regression analyses, but do indicate that further research is warranted to understand the unique elements in these parenting constructs and inform operationalization. The measure of parental control utilized in the current study combined behavioral, affective and cognitive aspects of parental control. The scale was formed of a collection of items taken from a broad-based measure of parenting, which combined to indicate parenting behavior that is controlling or restrictive of child autonomy (Borelli and Margolin, 2013, Unpublished). Some of the items in the scale seem to also overlap with other parenting constructs, such as parental rejection (e.g., ‘I am less friendly when my child doesn’t see things my way’). In the context of child anxiety, parental control has been referred to as vigilance, intrusion, and inhibition of the child’s independence (Bőgels and Brechman-Toussant, 2006). Thus, parental control can be considered as an over-involvement and restriction of the child’s autonomy. In this sense, it is expected that parental control will overlap to varying degrees with other parenting constructs. Measurement methods other than self-report will be essential to extend our understanding of where and when parental experiential avoidance and parental control diverge and meet in the context of childhood anxiety. Future research should therefore utilize observational tools of parenting or at a minimum incorporate measures with a clear operationalization of parental control.

The majority of participants in this study described themselves as white British and were mostly mothers from a community sample in one part of Northern England; therefore, the generalizability of the findings is limited. The findings also require replication with children at other developmental stages and with clinical populations (parent and child).

### Practical Implications

The present findings have a number of practical implications. Given the observed relationships between parental anxiety, parenting factors and child anxiety, preventative approaches could consider targeting parents who experience high levels of anxiety. For example, it may be helpful to target parents who are seeking treatment for their own anxiety in adult mental health services and provide parenting interventions that teach parents about the impact their behavior may have on the development or maintenance of anxiety in their child. The content from established parenting programs (such as ‘From Timid to Tiger’ for anxious children and their parents; Cartwright-Hatton et al., 2010) could be used to inform programs for anxious parents accessing adult mental health services.

The highlighted role of parental control in child anxiety suggests that if parents are able to reduce the amount of control they employ in their parenting; this may reduce the risk of anxiety in their child. In the treatment of anxiety in childhood, Cognitive Behavioral Therapy (CBT) has been identified as an empirically supported intervention (see James et al., 2013 for a meta-analytic review). Borelli et al. (2015) suggest that in order to work on reducing parental control, parents could be supported to use imaginal exposure or behavioral experiments to slowly reduce the amount of control they exert over their children. However, given that the current study has also found that the concept of parental experiential avoidance is an important predictor in child anxiety, it may be that these methods would be difficult for parents who struggle to tolerate their anxious child’s distress and consequently their own distress.

Parenting programs designed for parents of anxious children have focused on parental rearing behavior as reinforcers of child anxiety, such as parent modeling of fear response (Cartwright-Hatton et al., 2010). Some family-based therapies for child anxiety have also included a parenting component as an adjunct to individual child CBT. Parents’ own anxiety and their expectations of their child are targeted with CBT strategies (Ginsburg and Schlossberg, 2002). It may be a useful enhancement for such interventions to also target parental experiential avoidance first. Pertinent to this idea, when children receive CBT, a key component of the treatment is that they are exposed to situations that make them feel anxious (Barmish and Kendall, 2010). However, it is often the parents who may be involved in supporting their child to do this as being exposed to anxious situations often takes place between therapy sessions. Hence, parents are in effect, asked to support their child in experiencing distress during these intentionally anxiety provoking situations. Parents may receive training in delivering these aspects of therapy, at which point they could also be provided with support to manage their own emotional experience of this therapeutic task. If parents engage in experiential avoidance, it is likely that they may not tolerate supporting their child to do this and might either not do the home exercises with their child or behave in ways to rid themselves of their distress, which in turn may maintain their child’s anxiety. Tiwari et al. (2008) suggested that parents should be recruited as collaborators in CBT for child anxiety, and their own experiential avoidance monitored through their child’s exposure-based therapy. Mindful parenting interventions have been shown to target parental experiential avoidance (Emerson et al., unpublished), and may prove a useful adjunct to CBT for child anxiety. Therefore, addressing parental experiential avoidance should be an element in both the prevention and treatment of child anxiety.

## Author Contributions

L-ME contributed to the design of the study, analyzed data, and drafted the manuscript. CO contributed to the design of the study, collected data, conducted data analyses, and contributed to writing the manuscript. GR contributed to the design of the study and to the writing of the paper.

## Conflict of Interest Statement

The authors declare that the research was conducted in the absence of any commercial or financial relationships that could be construed as a potential conflict of interest.
